# Neutralizing antibodies for the prevention and treatment of COVID-19

**DOI:** 10.1038/s41423-021-00752-2

**Published:** 2021-09-08

**Authors:** Lanying Du, Yang Yang, Xiujuan Zhang

**Affiliations:** 1 Lindsley F. Kimball Research Institute, New York Blood Center, New York, NY USA; 2 Roy J. Carver Department of Biochemistry, Biophysics and Molecular Biology, Iowa State University, Ames, IA USA

**Keywords:** SARS-CoV-2, Spike protein, Neutralization, Monoclonal antibodies, COVID-19, Viral infection, Immunotherapy

## Abstract

Severe acute respiratory syndrome coronavirus-2 (SARS-CoV-2) initiates the infection process by binding to the viral cellular receptor angiotensin-converting enzyme 2 through the receptor-binding domain (RBD) in the S1 subunit of the viral spike (S) protein. This event is followed by virus–cell membrane fusion mediated by the S2 subunit, which allows virus entry into the host cell. Therefore, the SARS-CoV-2 S protein is a key therapeutic target, and prevention and treatment of coronavirus disease 2019 (COVID-19) have focused on the development of neutralizing monoclonal antibodies (nAbs) that target this protein. In this review, we summarize the nAbs targeting SARS-CoV-2 proteins that have been developed to date, with a focus on the N-terminal domain and RBD of the S protein. We also describe the roles that binding affinity, neutralizing activity, and protection provided by these nAbs play in the prevention and treatment of COVID-19 and discuss the potential to improve nAb efficiency against multiple SARS-CoV-2 variants. This review provides important information for the development of effective nAbs with broad-spectrum activity against current and future SARS-CoV-2 strains.

## Introduction

Coronavirus disease 2019 (COVID-19) is a newly emerged infectious disease first identified in December 2019. The major clinical features of COVID-19 include fever, shortness of breath, cough, headache, and fatigue, which can lead to severe pneumonia, lung injury, acute respiratory/multiorgan failure, and death [1–4]. A variety of risk factors, including older age, hypertension, obesity, diabetes mellitus, and cardiovascular comorbidities, are associated with COVID-19 [5–8]. As of July 16, 2021, the WHO had reported over 188.6 million confirmed COVID-19 cases and more than 4 million deaths worldwide, resulting in devastating damage. Although several vaccines, including two mRNA vaccines (BNT162b2 and mRNA-1273) and one adenovirus-based vaccine (Ad.26.COV2.S), have been authorized by the U.S. FDA for immunizing persons 16 years and older (BNT162b2) or 18 years (mRNA-1273) and older to prevent COVID-19 [9–11], effective countermeasures are still needed to control the global COVID-19 pandemic.

Severe acute respiratory syndrome coronavirus-2 (SARS-CoV-2) is the causative agent of COVID-19. It belongs to the *Betacoronavirus* genus of the *Coronaviridae* family in the order Nidovirales. SARS-CoV-1 and Middle East respiratory syndrome coronavirus (MERS-CoV), two other highly pathogenic coronaviruses first reported in 2002 and 2012, respectively [12, 13], are also betacoronaviruses. Both SARS-CoV-1 and MERS-CoV have limited transmission among humans, leading to case fatality rates of approximately 10% (774/8098) for SARS-CoV-1 [14] and approximately 34.4% (886/2574) for MERS-CoV. Compared to SARS-CoV-1 and MERS-CoV, SARS-CoV-2 exhibits superior human-to-human transmissibility [15–17], potentially contributing to its widespread infection and the global COVID-19 pandemic.

**Fig. 1:**
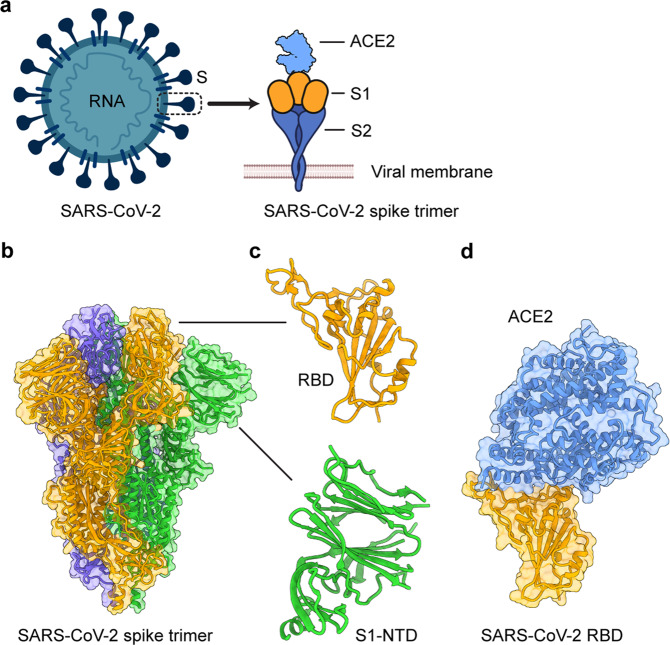
Structural overview of the SARS-CoV-2 spike (S) protein. **a** Schematic diagram of the SARS-CoV-2 virion and its S protein. E envelope, M membrane, N nucleocapsid, ACE2 angiotensin-converting enzyme 2. **b** Cryo-EM structure of the SARS-CoV-2 S protein trimer (PDB 6VXX). The three subunits are colored orange, green, and blue. **c** Close-up views of the SARS-CoV-2 S receptor-binding domain (RBD) and N-terminal domain (NTD) in the S1 subunit. **d** Crystal structure of the SARS-CoV-2 RBD in complex with human ACE2 (PDB 6M0J). Human ACE2 is colored in light blue

Similar to other coronaviruses, SARS-CoV-2 is an enveloped, single-stranded, and positive-sense RNA virus [18]. The viral genome encodes four major structural proteins (spike (S), membrane (M), envelope (E), and nucleocapsid (N)) (Fig. 1a), several nonstructural proteins (NSP1-16), and a series of accessory proteins, including 3a, 6, 7a, 7b, 8, and 10 [18, 19]. NSP proteins form replication-transcription complexes and mainly participate in biological processes, such as viral replication, protein processing, transcription, and proteolysis. Some function as RNA-dependent RNA polymerases, in addition to binding ATP and zinc ions [19, 20]. The E and M proteins may be involved in RNA packaging and virus assembly and release; the E protein has evolved to include a robust membrane topology, and it forms either a cation channel regulated by pH or an ion channel potentially inhibited by gliclazide and memantine [19, 21–23]. The N protein, which is located inside the virion, is responsible for RNA packaging and virus replication. It contains phosphorylation and protein kinase sites and might be important in modulating antiviral immunity and inhibiting interferon production by targeting the retinoic acid inducible gene-I-like receptor pathway [24–26].

## SARS-CoV-2 S protein receptor binding and membrane fusion

Similar to the S proteins of other pathogenic coronaviruses, the S protein of SARS-CoV-2 plays the most important role in virus infection and pathogenesis and is therefore an important target for designing and developing effective COVID-19 vaccines and therapeutic antibodies. The S protein contains the S1 and S2 subdomains (Fig. 1a, b), which are responsible for host cell receptor binding and membrane fusion, respectively, mediated through the receptor-binding domain (RBD) in the S1 region (Fig. 1c) and heptad repeat region 1 (HR1) and HR2 in the S2 region [27, 28]. The N-terminal domain (NTD) is located on the S1 subunit (Fig. 1c) and has the potential to bind sialic acids or coreceptors [29, 30], but its specific function is still not fully understood. The RBD is composed of a core and a receptor-binding motif (RBM), and the latter is responsible for binding to the SARS-CoV-2 receptor [28, 31]. Similar to SARS-CoV-1 and some SARS-like bat coronaviruses, such as W1V1-CoV, SHC014-CoV, and RaTG13, SARS-CoV-2 utilizes angiotensin-converting enzyme 2 (ACE2) as its functional cellular receptor to enter target cells to initiate the virus infection process [28, 32]. In addition to ACE2, SARS-CoV-2 also uses the serine protease TMPRSS2 for S protein priming [33]. Moreover, neuropilin-1, a cofactor for binding furin-cleaved substrates, facilitates SARS-CoV-2 cell entry and infectivity [34].

Cryo-EM and/or crystal structures of the SARS-CoV-2 S-trimer/ACE2 and RBD/ACE2 complexes have been resolved (Fig. 1d). The prefusion conformation of the SARS-CoV-2 S protein in cryo-EM analysis presents as a trimeric structure consisting of three RBDs, with one RBD in the “up” conformation, allowing access to the ACE2 receptor [35, 36]. ACE2 recognizes the RBD through polar residues in its extracellular peptidase domain [37]. Analysis of the crystal structure of the RBD/ACE2 complex has identified critical residues in the RBD that are essential for binding to the ACE2 receptor, eight of which are identical in the SARS-CoV-2 and SARS-CoV-1 RBDs; the RBM contains most of the amino acid residues necessary for binding to ACE2 [31]. Relative to the SARS-CoV-1 S RBD, the SARS-CoV-2 S RBD has a higher binding affinity for ACE2 [28, 35, 38], partially explaining the more efficient human-to-human transmission of SARS-CoV-2.

**Fig. 2:**
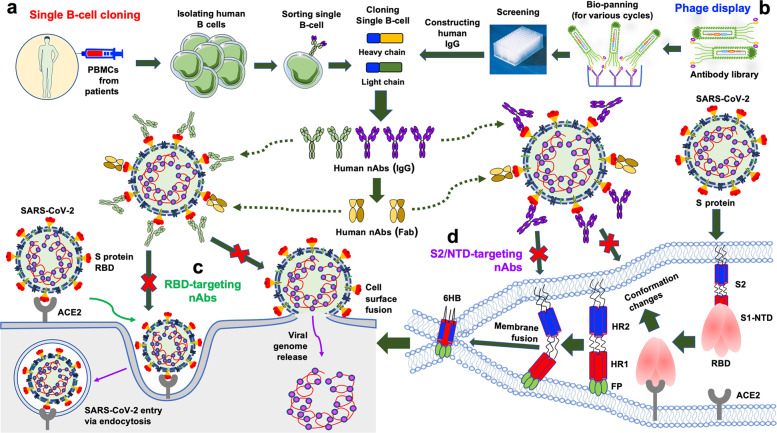
Generation of SARS-CoV-2-targeting human neutralizing monoclonal antibodies (nAbs) and their mechanisms of action. nAbs specific to SARS-CoV-2 can be generated by single B cell cloning (**a**) or phage display library screening (**b**). **c** nAbs targeting the receptor-binding domain (RBD) of the spike (S) protein bind to the RBD in the S1 subunit and block its binding with the angiotensin-converting enzyme 2 (ACE2) receptor, thus preventing virus entry into host cells through endocytosis or cell surface fusion processes. **d** nAbs targeting the N-terminal domain (NTD) in the S1 subunit or S2 bind to the NTD or S2 subunit, thus inhibiting the conformational change of the S protein or NTD or the formation of a 6-helix bundle (6-HB) structure mediated by heptad repeat region 1 (HR1) and HR2 in the S2 subunit, further blocking membrane fusion and virus entry into the host cell

During SARS-CoV-2 infection, the RBD in the S1 subunit of the S protein first binds the cellular receptor ACE2 to form an RBD/ACE2 complex (Figs 1 and 2). This binding event leads to conformational changes in the S protein and subsequent dissociation of the S1 and S2 subunits. Mediated by the HR1/HR2 domains in the S2 subunit, the viral and host cell membrane fusion process occurs, allowing the virus to enter the host cell and release viral RNA [39]. Subsequently, newly synthesized viral RNA–N complexes and S, E, and M proteins are further assembled in the endoplasmic reticulum–Golgi intermediate compartment to form mature virions, which are released from host cells [40]. Relative to SARS-CoV-1, SARS-CoV-2 demonstrates better membrane fusion ability, and changes in several amino acid residues in the HR1 domain potentially contribute to the increased protein–protein interactions with the HR2 domain [27]. A better understanding of the viral life cycle and associated infection process will be helpful for designing effective vaccines and therapeutic agents to control the COVID-19 pandemic.

## SARS-CoV-2-specific neutralizing monoclonal antibodies and their mechanisms of action

Neutralizing monoclonal antibodies (nAbs) serve as key therapeutic agents for the rapid prevention and treatment of SARS-CoV-2 infection. Anti–SARS-CoV-2 human nAbs can be isolated from antigen-specific B cells from the peripheral blood mononuclear cells of individuals who have been infected with SARS-CoV-2 (Fig. 2a). They can also be generated by screening naive or synthetic phage-displayed human antibody libraries (Fig. 2b) [41–51]. Most of these antibodies target the SARS-CoV-2 S protein, including its subdomains, and they present different mechanisms of action in the inhibition of SARS-CoV-2 infection.

Generally, nAbs targeting the S protein block receptor binding or membrane fusion, thereby preventing viral entry into host cells. Specifically, RBD-targeting nAbs bind to the RBD in the RBM region and inhibit RBD binding to the ACE2 receptor, thus blocking the subsequent viral entry process (Fig. 2c). Some non-ACE2 mimics target the RBD without inhibiting RBD-ACE2 binding and may instead inhibit SARS-CoV-2 infection by blocking S protein conformational changes. NTD-targeting nAbs bind to the NTD of the S protein, and the recognition sites may contain N-glycosylation sites [52, 53]. They neutralize SARS-CoV-2 infection by potentially preventing the interaction of the NTD with C-type lectin receptors, such as L-SIGN, inhibiting S protein or NTD conformational changes, or interrupting virus postattachment steps (cell–cell fusion) (Fig. 2d) [41, 52, 54–56]. However, these nAbs do not generally block the receptor binding and viral attachment steps. In contrast to NTD-specific nAbs that neutralize SARS-CoV-2 infection, some NTD-targeting non-nAbs can induce the open conformation of the RBD, resulting in enhanced binding of the RBD to the ACE2 receptor and thus increasing SARS-CoV-2 infectivity [57]. Both NTD- and RBD-targeting nAbs may activate Fc-mediated effector functions, antibody-dependent cellular phagocytosis, or antibody-dependent cellular cytotoxicity, which are potentially required for optimal protection against SARS-CoV-2 challenge [41, 54, 58, 59]. S2-targeting nAbs may inhibit HR1 or HR2 from forming a six-helix bundle structure, thereby blocking subsequent membrane fusion and the viral entry process (Fig. 2d).

## Antigenic view of the NTD and RBD of SARS-CoV-2 S protein and nAb binding sites

### Antigenic view of SARS-CoV-2 NTD and nAb binding sites

The NTD of the SARS-CoV-2 S protein is an immunodominant target to induce the production of neutralizing antibodies, although the neutralizing activity of nAbs targeting the NTD is generally much lower than that of the nAbs targeting the RBD. Antigenic and/or epitope mapping of the NTD has identified several supersites of SARS-CoV-2 recognized by NTD-specific nAbs [41, 52]. NTD-specific nAbs can be categorized into two distinct groups: a group with high potency against SARS-CoV-2 infection and a group with less potency but displaying glycan-dependent neutralizing activity [53].

### Antigenic view of SARS-CoV-2 RBD and nAb binding sites

The RBD of the SARS-CoV-2 S protein is an immunodominant target for inducing the production of highly potent and specific neutralizing antibodies. Analysis of the binding between the RBD and RBD-specific nAbs, as well as the inhibition of RBD-ACE2 binding by nAbs, reveals different antigenic regions on the RBD. These regions can be clustered based on the site for antibody attachment that overlaps the ACE2 binding site, as well as the antibody binding site that does not compete with ACE2 binding [60]. Nevertheless, the N and C termini of the RBD generally have less ability to bind nAbs [60]. Accordingly, RBD-targeting nAbs are classified into different groups based on the recognized epitopes on the RBD. These groups include nAbs binding to the RBD site distal to the ACE2 receptor-binding site (normally having low neutralizing activity) and nAbs directly competing with the ACE2 binding site at the RBM region (generally presenting the most potent neutralizing activity); other RBD-specific nAbs recognize epitopes partially overlapping with or further distal from the above sites [53].

Following the COVID-19 pandemic, an increasing number of human nAbs targeting SARS-CoV-2 proteins were identified and developed as therapeutics. The majority of these nAbs target different regions of the S protein, including the NTD and RBD, whereas a few antibodies target other proteins. In the rest of this review, we will summarize the SARS-CoV-2-specific nAbs currently developed based on the domains they target in the viral proteins.

### Neutralizing antibodies targeting the NTD of SARS-CoV-2 S protein

As part of the S1 domain of the S protein, the NTD is a key target for the development of COVID-19 nAbs, and several of the currently developed human nAbs bind to this region. These NTD-targeting nAbs may neutralize SARS-CoV-2 infection in vitro or protect animals from viral challenge (Table 1) [41, 44, 54, 55, 61]. However, it appears that none of these NTD-targeting nAbs have been tested in humans.

**Table 1:** Representative human neutralizing monoclonal antibodies (nAbs) against SARS-CoV-2

Name	Binding affinity	Neutralization against SARS-CoV-2 infection	Protection from wild-type SARS-CoV-2 infection	References
*nAbs targeting the NTD of SARS-CoV-2*				
4-8 5-7 2-17 4-18 5-24	Bound to SARS-CoV-2 S trimer and NTD proteins	Neutralized infection by wild-type pseudotyped (IC_50_ ≤ 168 ng/ml) and live (IC_50_ ≤ 33 ng/ml) SARS-CoV-2	N/A	[44]
4A8	Bound to SARS-CoV-2 S (K_D_ 0.996 nM) and S1 (K_D_ 92.7 nM) proteins	Neutralized infection by wild-type pseudotyped (EC_50_ 49 μg/ml) and live (EC_50_ 0.61 μg/ml) SARS-CoV-2	N/A	[55]
89C8	Bound to SARS-CoV-2 NTD protein	Neutralized pseudotyped SARS-CoV-2 infection (IC_50_ 4.5 nM)	N/A	[61]
S2L28 S2M28 S2X28 S2X333	Bound to SARS-CoV-2 and bat RaTG13 S protein NTDs (EC_50_ < 100 ng/ml); binding to SARS-CoV-2 NTD escape or natural mutants was reduced	Neutralized infection by wild-type pseudotyped (IC_50_ < 50 ng/ml) and live SARS-CoV-2 (IC_50_ 2–29 ng/ml); neutralization of mutant SARS-CoV-2 was reduced; neutralized bat RaTG13 pseudovirus infection	Prophylactically protected hamsters from SARS-CoV-2 challenge (for S2X333), with reduced viral replication and/or titers in the lung	[41]
COV2-2489 COV2-2676	Bound to a common antigenic site in the SARS-CoV-2 NTD	Neutralized infection by wild-type pseudotyped (IC_50_ 38 or 58 ng/ml) and live (IC_50_ 501 or 199 ng/ml) SARS-CoV-2	Prophylactically and therapeutically protected hACE2-Tg mice from SARS-CoV-2 infection, with reduced weight loss, viral titers, cytokine levels, or chemokine levels	[42, 54]
*nAbs targeting the RBD of SARS-CoV-2*				
P5A-1B8 P5A-1B9 P5A-2G7 P5A-3C12	Bound to the SARS-CoV-2 RBD (K_D_ 0.75–3.55 nM), competing with ACE2 binding	Neutralized infection by wild-type pseudotyped (IC_50_ 0.01–0.66 nM) and live (IC_50_ 0.03–1.76 nM) SARS-CoV-2	N/A	[63]
TAU-2189 TAU-2230 TAU-2303 TAU-1109	Bound to the SARS-CoV-2 RBD with or without blocking RBD-ACE2 binding	Neutralized infection by wild-type pseudotyped (IC_50_ 0.05–1.05 μg/ml) and live (IC_50_ 10 μg/ml) SARS-CoV-2	N/A	[45]
MD45 MD47 MD62 MD65 MD67	Bound to SARS-CoV-2 S, S1, and the RBD (K_D_ 0.5–5.8 nM) with or without blocking RBD-ACE2 binding	Neutralized infection of wild-type live (IC_50_ 0.22–13 μg/ml) SARS-CoV-2	N/A	[48]
COVA1-18 COVA2-15	Bound to SARS-CoV-2 S and the RBD, competing with ACE2 binding	Neutralized infection of wild-type pseudotyped (IC_50_ 8 ng/ml) and live (IC_50_ ≤ 9 ng/ml) SARS-CoV-2	N/A	[43]
IgG1 ab1	Bound to SARS-CoV-2 S, S1, and the RBD (K_D_ 0.16 nM), competing the binding of the RBD with ACE2	Neutralized infection of wild-type pseudotyped (IC_50_ 200 ng/ml) and live (IC_50_ 100 ng/ml) SARS-CoV-2	Prophylactically and/or therapeutically protected wild-type mice from mouse-adapted SARS-CoV-2 infection or hACE2-Tg mice and hamsters from wild-type SARS-CoV-2 infection, with reduced viral titers or replication in the lung, nasal washes, and oral swabs (for hamsters)	[59]
COV2-2196 COV2-2130 COV2-2381	Bound to SARS-CoV-2 S trimer and the RBD (EC_50_ 0.1–10 ng/ml), fully blocking RBD-ACE2 binding	Neutralized infection by wild-type pseudotyped (IC_50_ ≤ 110 ng/ml) and live (IC_50_ ≤ 107 ng/ml) SARS-CoV-2, showing synergistic effects (for COV2-2196 and COV2-2130)	Prophylactically and/or therapeutically protected AdV-hACE2-transduced mice from SARS-CoV-2 infection or wild-type mice from mouse-adapted SARS-CoV-2 infection, with reduced weight loss, viral replication or inflammation; prophylactically protected NHPs from SARS-CoV-2 replication	[42, 75]
CV07-209	Bound to the SARS-CoV-2 RBD (K_D_ 0.006 nM; EC_50_ 4.1 ng/ml), blocking RBD-ACE2 attachment	Neutralized infection by wild-type live SARS-CoV-2 (IC_50_ 3.1 ng/ml)	Prophylactically and therapeutically protected hamsters from SARS-CoV-2 infection, preventing weight loss and lung pathology	[64]
CC6.29 CC6.30 CC12.1	Bound to SARS-CoV-2 S and the RBD, competing with ACE2 binding	Neutralized infection by wild-type (IC_50_ 1–19 ng/ml) or V367F, G476S, and D614G variants of pseudotyped SARS-CoV-2	CC12.1 prophylactically protected hamsters from SARS-CoV-2 challenge, with reduced weight loss and viral loads	[73]
2-4 2-7 1-20 1-57 2-15	Bound to the SARS-CoV-2 S trimer and RBD proteins	Neutralized infection by wild-type pseudotyped (IC_50_ ≤ 394 ng/ml) and live (IC_50_ ≤ 57 ng/ml) SARS-CoV-2	Prophylactically protected hamsters from SARS-CoV-2 infection, with reduced viral titers and replication in the lung	[44]
J08 I14 F05 G12 C14 B07	Bound to the SARS-CoV-2 S trimer, S1, and RBD proteins with high potency	Neutralized infection by wild-type, D614G, and B.1.1.7. variants (IC_50_ 3.9–157.5 ng/ml) of live SARS-CoV-2	An Fc-engineered version of J08 (J08-MUT) prophylactically and therapeutically protected hamsters from SARS-CoV-2 infection in a dose-dependent manner, without weight loss or with reduced weight loss or viral titers in the lung	[62]
MV05	Bound to prototypic (K_D_ 0.403 nM) and mutant SARS-CoV-2 RBDs, disrupting RBD-ACE2 binding	Neutralized infection by wild-type pseudotyped (IC_50_ 0.03 μg/ml) and live (IC_50_ 1 μg/ml) SARS-CoV-2	An Fc-engineered version (MV05/LALA) prophylactically and therapeutically protected NHPs from SARS-CoV-2 infection, preventing weight loss and viral replication	[74]
CB6	Bound to the SARS-CoV-2 RBD (K_D_ 2.49 nM), blocking its binding with ACE2	Neutralized infection by wild-type pseudotyped (IC_50_ 23 ng/ml) and live (IC_50_ 36 ng/ml) SARS-CoV-2	An Fc-engineered version (CB6(LALA)) prophylactically and therapeutically protected NHPs from SARS-CoV-2 infection, with reduced viral titers and limited lung damage	[65]
CT-P59	Bound to the SARS-CoV-2 RBD (K_D_ 0.027 nM) protein, completely blocking its binding with ACE2	Neutralized infection by wild-type (IC_50_ 8.4 ng/ml) and D614G variant (IC_50_ 5.7 ng/ml) live SARS-CoV-2	Therapeutically protected ferrets, hamsters, and NHPs from SARS-CoV-2 infection, with reduced viral titers and clinical symptoms	[47]
*nAbs targeting other regions of the S protein or S trimer of SARS-CoV-2*				
2-43 2-51	Bound to SARS-CoV-2 non-NTD and non-RBD S protein regions	Neutralized infection by wild-type pseudotyped (IC_50_ 71 or 652 ng/ml) and live SARS-CoV-2 (IC_50_ 3 or 7 ng/ml)	N/A	[44]
I21 J13 D14	Bound to SARS-CoV-2 non-RBD S1 protein region	Neutralized infection by wild-type, D614G, and/or B.1.1.7 (IC_50_ 99.2–500 ng/ml) variants of live SARS-CoV-2	N/A	[62]
0304-3H3 9A1	Bound to SARS-CoV-2 S (K_D_ ≤ 2.14 nM) and S2 (K_D_ ≤ 4.52 nM) proteins	Showed moderate-to-no neutralization against infection by wild-type pseudotyped or live SARS-CoV-2	N/A	[55]
L19	Bound to SARS-CoV-2 S trimer and S2 proteins	Showed low neutralization potency against infection by wild-type (19.8 μg/ml), D614G (IC_50_ 12.5 μg/ml), and B.1.1.7 (9.9 μg/ml) variants of live SARS-CoV-2	N/A	[62]
H20 I15 F10 F20	Bound to SARS-CoV-2 S trimer with low affinity	Showed moderate neutralization potency (IC_50_ 155–492.2 ng/ml) against infection by wild-type and D614G mutant live SARS-CoV-2	N/A	[62]
*Multimeric nAbs targeting multiple SARS-CoV-2 proteins*				
89C8-ACE2	Bound to SARS-CoV-2 NTD and RBD proteins	Inhibited S1–ACE2 interaction, preventing infection by wild-type pseudotyped (IC_50_ 29 nM) and live (1.7 nM) SARS-CoV-2	N/A	[61]

*ACE2* angiotensin-converting enzyme 2, *AdV-hACE2* adenovirus-expressing hACE2, *EC*_*50*_ half-maximal effective concentration, *hACE2-Tg* human ACE2 transgenic, *IC*_*50*_ half-maximal inhibitory concentration, *K*_*D*_ equilibrium dissociation constant, *nAb* neutralizing monoclonal antibody, *NHP* nonhuman primate, *NTD* N-terminal domain, *RBD* receptor-binding domain

#### Binding ability of SARS-CoV-2 NTD-targeting nAbs

**Fig. 3:**
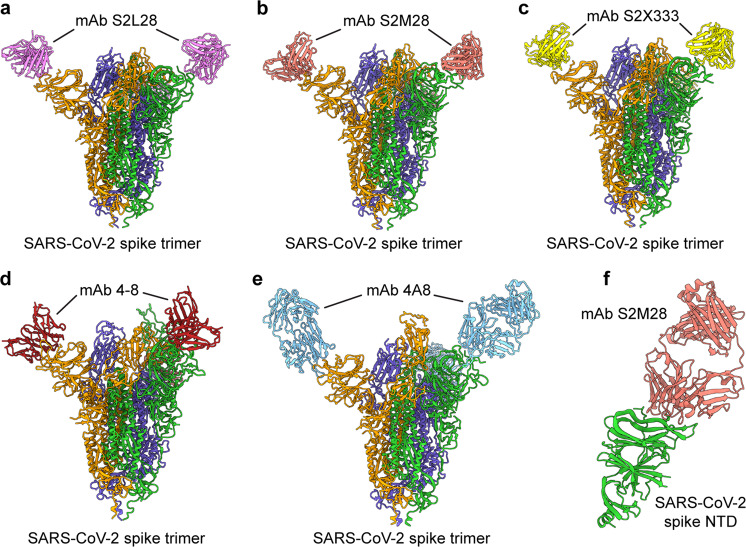
Structures of SARS-CoV-2 spike (S) in complex with N-terminal domain (NTD)-targeting neutralizing monoclonal antibodies (nAbs). **a**–**e** Cryo-EM structures of the SARS-CoV-2 S trimer bound to NTD-targeting nAbs **a** S2L28 (PDB 7LXZ), **b** S2M28 (PDB 7LY2), **c** S2X333 (PDB 7LXY), **d** 4-8 (PDB 7LQV), and **e** 4A8 (PDB 7C2L). **f** Crystal structure of the SARS-CoV-2 NTD in complex with the nAb S2M28 (PDB 7LY3)

NTD-targeting nAbs bind to the SARS-CoV-2 NTD and S1 fragments of the S protein and/or S protein trimer. Cryo-EM and crystal structure analyses have identified critical neutralizing epitopes for several SARS-CoV-2 NTD-targeting nAbs (Fig. 3a–f) [41, 44, 55, 56]. For example, cryo-EM and crystal structures of the S2L28/S, S2M28/S, S2X28/S, S2X333/S, and S2M28/NTD complexes reveal that these nAbs recognize overlapping epitopes within a structurally identified antigenic supersite in the NTD of the S protein [41]. In addition, cryo-EM and/or crystal structures of NTD-specific nAbs, such as 5-24, 1-87, and 2-51, in complex with S or the NTD reveal a single supersite surrounded by glycans at N17, N74, N122, and N149, which is located at the periphery of S facing away from the viral membrane [56]. Thus, structural analysis of nAb/S and nAb/NTD complexes is helpful for determining which residues are important for nAb binding to the NTD of S and identifying neutralizing epitopes recognized by these nAbs.

#### Neutralizing activity of SARS-CoV-2 NTD-targeting nAbs

SARS-CoV-2 NTD-targeting nAbs can neutralize wild-type pseudotyped and/or authentic SARS-CoV-2 infection in vitro. Pseudotyped and authentic SARS-CoV-2 have been found to be neutralized by nAbs 4-8 and 5-24 with a half-maximal inhibitory concentration (IC_50_) of 8–9 ng/ml [44]. nAb 4A8 also has been shown to neutralize both pseudovirus and live SARS-CoV-2 infection with a half-maximal effective concentration (EC_50_) of 0.61 and 49 μg/ml, respectively [55]. 89C8 has been found to neutralize infection of pseudotyped SARS-CoV-2, with an IC_50_ of 4.5 nM [61], whereas nAbs BLN1, BLN12, and P008_056 have been shown to neutralize authentic SARS-CoV-2 infection with IC_50_ values of 8, 8, and 14 ng/ml, respectively [52, 53]. Moreover, S2L28, S2M28, S2X28, and S2X333 have been found to potently neutralize infection of both pseudotyped and live SARS-CoV-2 with an IC_50_ value as low as 2 ng/ml [41]. The differences in potency of these nAbs against SARS-CoV-2 infection might result in part from differences in the neutralization assays and virus strains used for detecting neutralizing activity.

#### Protective efficacy of SARS-CoV-2 NTD-targeting nAbs

Several NTD-targeting nAbs have demonstrated prophylactic and therapeutic protective efficacy against SARS-CoV-2 infection in animal models, such as hACE2-transgenic (hACE2-Tg) mice and hamsters [41, 54]. For example, COV2-2676 and COV2-2489, which bind to a common antigenic site on the NTD and inhibit pseudotyped and authentic SARS-CoV-2 infection in vitro, have been found to prophylactically and therapeutically protect hACE2-Tg mice from SARS-CoV-2 infection. These animals exhibited reduced weight loss, decreased viral titers in the upper and lower respiratory tracts and heart, or reduced cytokine and chemokine levels in the lung [54]. NTD-targeting nAbs 159, BLN12, and BLN14 have been shown to therapeutically protect against SARS-CoV-2 infection in antibody-treated hACE2-Tg mice, preventing weight loss, with reduced viral titers and/or RNA copies in the nasal washes, lung, and other tissues [52, 60]. Moreover, S2X333 has been found to prophylactically protect hamsters from SARS-CoV-2 infection; these animals had reduced viral RNA copies and/or viral titers in the lung [41].

#### Cross-reactivity and cross-neutralizing activity of SARS-CoV-2 NTD-targeting nAbs

SARS-CoV-2 NTD-targeting nAbs may cross-react with other coronaviruses and/or cross-neutralize their infection to some extent. This phenomenon is observed for S2L28, S2M28, S2X28, and S2X333, which strongly bind to the S protein of RaTG13 (a bat coronavirus most closely related to SARS-CoV-2) and neutralize infection by pseudotyped RaTG13 expressing the viral S protein. However, their ability to bind to other coronavirus S proteins, such as those from Pangolin Guangxi 2017 and Pangolin Guangdong 2019, is reduced, and none of them neutralize infection by human SARS-CoV-1, Pangolin Guangxi 2017, Pangolin Guangdong 2019, bat W1V1, or bat W1V16 pseudoviruses [41]. These differences in binding affinity and subsequent viral neutralization may result from sequence variation within the S protein NTD regions for each coronavirus.

### Neutralizing antibodies targeting the RBD of SARS-CoV-2 S protein

The SARS-CoV-2 S protein RBD is a critical target for the development of effective COVID-19 antibodies. SARS-CoV-2 RBD-targeting nAbs have been extensively studied, and most of the currently developed human nAbs are specific to this region. These nAbs bind to the RBD of SARS-CoV-2 S, neutralize SARS-CoV-2 infection in vitro, and/or protect animals from SARS-CoV-2 challenge (Table 1). However, most of these nAbs are in preclinical development, and only a few have progressed into human clinical trials [62].

#### Binding and cross-reactivity of SARS-CoV-2 RBD-targeting nAbs

RBD-targeting SARS-CoV-2 nAbs generally have high binding affinities to the RBD of SARS-CoV-2 S. Most of the RBD-targeting nAbs, including P2C-1F11, P2B-2F6, P2C-IA3, P5A-1B8, P5A-2G7, MD62, MD65, COVA1-18, COVA2-15, IgG1 ab1, CV07-209, MV05, CA1, CB6, CT-P59, Ab6, ab8, V_H_-Fc ab8, m397, and 1212C2, bind to the ACE2-binding region of the RBD, directly blocking the binding interaction between the RBD and the cellular receptor ACE2 [43, 47–49, 59, 63–68]. Other nAbs, including TAU-1109, MD29, and MD47, strongly bind to the RBD but at regions distal to the ACE2 binding site; hence, they do not compete with ACE2 for RBD binding [48]. Only a few SARS-CoV-2 RBD-specific nAbs, such as CV38-142, COVA1-07, COVA1-16, COVA2-02, COVA2-44, and CoV2-12, cross-react against SARS-CoV-1 S and/or RBD proteins, potentially because they recognize conserved epitopes within the SARS-CoV-2 and SARS-CoV-1 RBDs [43, 51, 64].

**Fig. 4:**
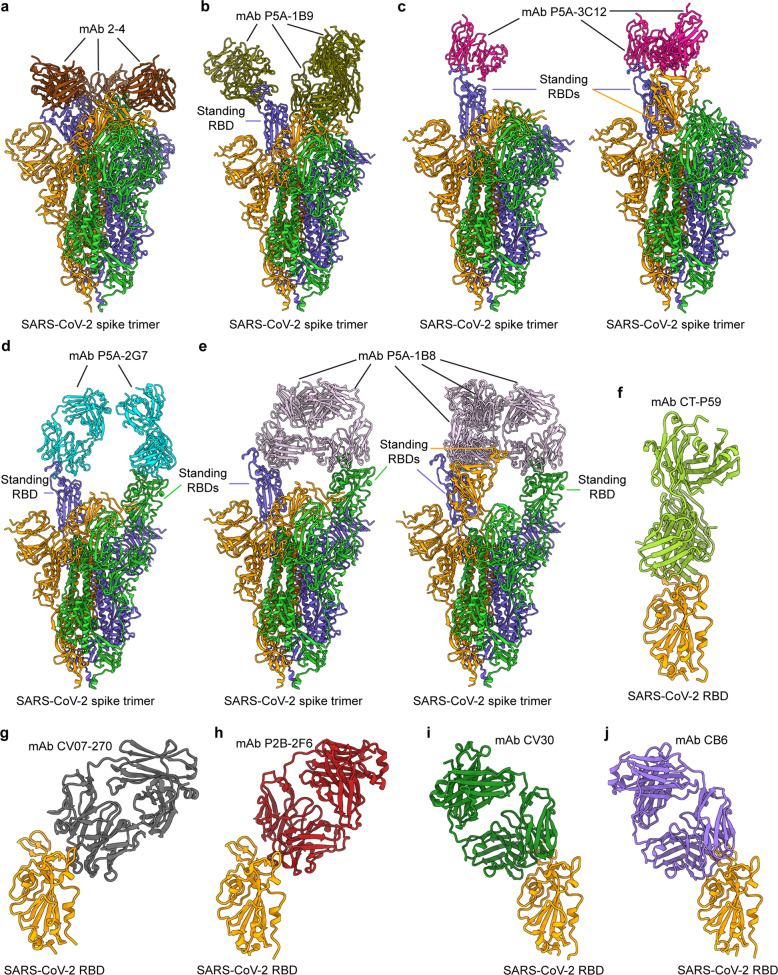
Structures of SARS-CoV-2 spike (S) in complex with receptor-binding domain (RBD)-targeting monoclonal antibodies (mAbs) with neutralizing activity (nAbs). **a**–**e** Cryo-EM structures of the SARS-CoV-2 S trimer bound to nAbs. **a** 2-4 with all three RBDs in the down conformation (PDB 6XEY), **b** P5A-1B9 with one RBD in the up conformation (PDB 7CZX), **c** P5A-3C12 with one or two RBDs in the up conformation, respectively (PDBs 7D0B and 7D0D), **d** P5A-2G7 with two RBDs in the up conformation (PDB 7CZW), and **e** P5A-1B8 with two or three RBDs in the up conformation, respectively (PDBs 7CZR and 7CZS). **f**–**j** Crystal structures of the SARS-CoV-2 RBD in complex with the nAbs **f** CT-P59 (PDB 7CM4), **g** CV07-270 (PDB 6XKP), **h** P2B-2F6 (PDB 7BWJ), **i** CV30 (PDB 6XE1), and **j** CB6 (PDB 7C01)

Cryo-EM and crystal structures are available for several SARS-CoV-2 RBD-targeting nAbs in complex with the SARS-CoV-2 S protein RBD (Fig. 4) [44, 47, 49, 64, 65, 69]. Cryo-EM structures of the nAb Fab or IgG and SARS-CoV-2 S trimer complexes reveal that the binding of nAbs with the three RBDs can result in multiple conformations: three “down” RBDs, one “up” and two “down” RBDs, one or two “up” RBDs, two “up” RBDs, or two or three “up” RBDs (Fig. 4a–e) [44, 63]. Relative to the Fab nAb, the IgG nAb exhibits more potency in neutralizing SARS-CoV-2 infection [63]. Crystal structures of CT-P59, CV07-250, or CV07-270 (Fab/IgG) and RBD complexes show that CT-P59 and CV07-250 directly interact with or obscure the ACE2 binding sites, while CV07-270 only partially overlaps with these binding sites (Fig. 4f–g) [47, 64]. Crystal analysis of the P2B-2F6 Fab/RBD complex reveals that the epitopes recognized by the nAb are in the RBM region (Fig. 4h), demonstrating that the blockage of viral entry by this nAb is achieved through inhibiting ACE2 binding [49]. The crystal structure of the CV30 Fab/RBD complex also indicates that CV30 binds to epitopes necessary for ACE2 receptor binding (Fig. 4i) [69]. A crystal structure of the CB6 Fab/RBD complex reveals an epitope on the SARS-CoV-2 S protein RBD that overlaps with the ACE2 binding sites (Fig. 4j) [65]. Accordingly, structural analyses of the binding interactions between human nAbs and the SARS-CoV-2 S protein and/or RBD can map the common neutralizing epitopes recognized by these nAbs within the RBD, reveal the similarities between nAb/RBD binding and ACE2/RBD binding, and explain the differences, if any, between these antibodies in competing with the ACE2 receptor [64, 70–72]. These structures also help to elucidate the mechanisms by which these nAbs neutralize SARS-CoV-2 infection.

#### Neutralizing and cross-neutralizing activity of SARS-CoV-2 RBD-targeting nAbs

Most, if not all, nAbs specific to the SARS-CoV-2 S protein RBD potently neutralize wild-type SARS-CoV-2 pseudovirus and/or live virus infection in vitro, and they present more potent neutralizing activity than nAbs targeting non-RBD epitopes. These nAbs, including COVA1-18, COVA2-15, CV07-209, CC6.29, CC6.30, CC12.1, P008_108, 1212C2, and V_H_-Fc ab8, have been found to neutralize pseudovirus and/or live SARS-CoV-2 infection with high potency (IC_50_ ≤ 40 ng/ml) [43, 53, 64, 67, 68, 73]. The TAU-1145, TAU-2189, TAU-2230, and TAU-2303 nAbs, which recognize a key residue (G466) in the RBD, have been shown to neutralize both pseudotyped and live SARS-CoV-2 infection, inhibiting cell death or syncytium formation [45]. It has been noted that some RBD-targeting nAbs also neutralize infection by mutant variants of SARS-CoV-2 but at different potencies. CT-59 has been found to neutralize wild-type SARS-CoV-2 and the D614G mutant with IC_50_ values of 8.4 and 5.7 ng/ml, respectively [47]. Other nAbs, such as J08, I14, F05, G12, C14, and B07, have been shown to neutralize D614G and B.1.1.7 variants to different extents, and J08 has been found to also neutralize the E484K variant, albeit with relatively lower neutralizing activity [62]. Among the identified SARS-CoV-2 RBD-targeting nAbs, CC6.33, COVA2-02, and COVA1-16 also neutralize SARS-CoV-1 but at relatively high IC_50_ values of 162, 610, and 2,500 ng/ml, respectively [43, 73]. Most other SARS-CoV-2 RBD-targeting nAbs appear to have low or no cross-neutralizing activity against infection with SARS-CoV-1, MERS-CoV, or other coronaviruses [49].

#### Protective efficacy of SARS-CoV-2 RBD-targeting nAbs

A number of SARS-CoV-2 RBD-specific nAbs have demonstrated protective efficacy in different animal models, including mice (wild-type mice, adenovirus-hACE2 (AdV-hACE2)-transduced mice, and hACE2-Tg mice), hamsters, ferrets, and nonhuman primates (NHPs) [44, 47, 59, 64, 67, 68, 74–76]. RBD-targeting nAbs without Fc engineering protect against SARS-CoV-2 infection in vivo. For instance, IgG ab1 and V_H_-Fc ab8 have been found to prophylactically protect wild-type mice from infection with mouse-adapted SARS-CoV-2 and/or hACE2-Tg mice from infection with authentic SARS-CoV-2 [51, 59, 67]. IgG ab1, V_H_-Fc ab8, 1212C2, CV07-209, and CC12.1 have been shown to prophylactically and/or therapeutically protect hamsters from wild-type SARS-CoV-2 infection [59, 64, 67, 68, 73]. CT-59 has been found to exhibit therapeutic efficacy in preventing authentic SARS-CoV-2 infection in ferret, hamster, and NHP models, whereas 2-15, COV2-2196, and COV2-2381 have been shown to prophylactically protect hamsters or rhesus macaques from SARS-CoV-2 infection by inhibiting viral replication [44, 47, 75]. It has been reported that Fc-engineered SARS-CoV-2 RBD-targeting nAbs also provide protection against SARS-CoV-2 infection in vivo. For example, J08-MUT, which contains an Fc-engineered fragment of J08, has been found to prophylactically and therapeutically protect hamsters from wild-type SARS-CoV-2 infection [62]. In addition, the Fc-engineered MV05 and CB6 nAbs, with LALA mutations in the Fc domain, namely, MV05-LALA and CB6(LALA), respectively, have been shown to prophylactically and therapeutically protect NHPs from SARS-CoV-2 viral challenge [65, 74]. In addition to using the intraperitoneal (I.P.) route and parenteral routes, including intramuscular, subcutaneous, and intravenous injections, SARS-CoV-2 RBD-targeting nAbs can be administered via inhalation without reducing protective efficacy [47, 59, 62, 68, 73, 74]. For example, inhalation administration of Fc-engineered 1212C2 has been shown to eliminate viral loads in the lungs of SARS-CoV-2-challenged hamsters, resulting in better efficiency than injection via the I.P. route [68]. It is worth noting that the effective concentrations of nAbs required for the protection of animals against SARS-CoV-2 infection vary depending on the differences in nAb potency, viral titers, and strains and the animal models used for the challenge. It has been reported that serum concentrations of ~22 and 12 μg/ml (pseudovirus neutralization IC_50_×1160 and 630, respectively) of CC12.1 nAb protect hamsters challenged with SARS-CoV-2 (USA-WA1/2020 strain, 1 × 10^6^ plaque-forming units) from weight loss (100% or 50%, respectively) [73]. It appears that the neutralization potency of nAbs in vitro does not always correlate well with their protective efficacy in vivo [77]. This discrepancy might be because the Fc effector function of nAbs affects their ability to neutralize SARS-CoV-2 in vivo; thus, identification of optimal nAb concentrations in monotherapy or combinatorial therapy is needed to maximize nAb protection against SARS-CoV-2 infection [77].

#### Combination treatments with SARS-CoV-2 RBD-targeting nAbs

Combination treatments with SARS-CoV-2 RBD-targeting nAbs have exhibited synergistic effects in preventing infection by escape mutants or improving their neutralization in vitro and/or protecting against SARS-CoV-2 infection in animal models. In vitro, a cocktail of CoV2-06 and CoV2-14 has been found to effectively prevent SARS-CoV-2 infection by escape mutants, but neither nAb had this effect individually [51]. The combination of TAU-2212 (which recognizes an unknown conformational epitope on the S protein) with an ACE2-competing, RBD-binding nAb (such as TAU-2230, TAU-2189, TAU-1145, or TAU-2303), and a non-ACE2-competing/RBD-targeting nAb (TAU-1099) has been shown to improve its efficiency in inhibiting SARS-CoV-2 infection [45]. Furthermore, the RBD-targeting nAbs COV2-2196 and COV2-2130, which block RBD-ACE2 binding but recognize the nonoverlapping sites on the RBD of SARS-CoV-2, have been shown to synergistically neutralize SARS-CoV-2 infection with improved neutralizing activity compared with each of the two nAbs alone [75]. In vivo, treatment with COV2-2196 and COV2-2130, individually or in combination, has been found to prophylactically protect AdV-hACE2–transduced mice from infection with wild-type SARS-CoV-2; these mice exhibited no weight loss and had reduced viral titers and inflammation in the lung. Prophylactic and therapeutic combined treatment with COV2-2196 and COV2-2130 has been shown to improve protection against authentic SARS-CoV-2 infection in AdV-hACE2-transduced mice and protect wild-type mice from infection by a mouse-adapted SARS-CoV-2 variant [75]. Hamsters and NHPs treated prophylactically or therapeutically with REGN-COV2, a combination of REGN10987 and REGN10933, exhibit a reduction in viral load and pathology [78]. Despite these promising results, the combinatorial concentrations of nAbs required for in vivo protection against SARS-CoV-2 infection still need to be optimized for individual nAbs.

### Neutralizing antibodies targeting other regions of the SARS-CoV-2 S protein

Although a large number of nAbs identified to date target the SARS-CoV-2 RBD and the NTD, several nAbs target other regions (Table 1) [44, 55]. SARS-CoV-2 S2-targeting antibodies have been identified; however, the majority of them have no neutralizing activity. For example, among the 0304-3H3, 9A1, L19, P008_004, P008_005, P008_006, P008_016, P008_023, and P008_032 antibodies that bind to the SARS-CoV-2 S2 fragment, only L19 neutralizes infection by SARS-CoV-2, including variant strains (D614G and B.1.1.7) and the wild-type strain [53, 55, 62]. Several of these SARS-CoV-2 S2-targeting antibodies also cross-react with SARS-CoV-1 S protein, but none of them show neutralizing activity against SARS-CoV-1 infection [53]. In addition, nAbs binding to the S-trimer and other regions have been identified. For instance, the nAbs 2-43 and 2-51 bind to the SARS-CoV-2 S-trimer complex, recognizing non-NTD and non-RBD epitope(s) [44]. Other nAbs (such as I21, J13, D14, I15, F10, F20, and P008_060) bind to the S-trimer complex and/or non-RBD S1 proteins and are capable of neutralizing wild-type and/or the aforementioned variants in vitro [53, 62]. In general, the binding affinity and neutralizing activity of non-RBD and non-NTD epitope-specific nAbs against SARS-CoV-2 infection are lower than those of nAbs targeting the S protein NTD or RBD of SARS-CoV-2. It appears that no nAbs targeting non-NTD or non-RBD regions have yet been tested for protection against SARS-CoV-2 infection in vivo. It is worth noting that the structures of a few nAbs targeting the non-NTD and non-RBD regions of the SARS-CoV-2 S protein have been solved [44]. Identification of neutralizing epitopes outside the S protein NTD and RBD regions of SARS-CoV-2 is expected to lead to the design of novel and effective COVID-19 therapeutics and vaccines based on these epitopes.

### Neutralizing antibodies targeting other SARS-CoV-2 proteins

Only a few monoclonal antibodies developed to date target other proteins of SARS-CoV-2, and most do not have neutralizing activity against SARS-CoV-2 infection. However, they can be used for other applications, such as diagnosis and epidemiology. For example, monoclonal antibodies specifically targeting the SARS-CoV-2 N protein increase the detection sensitivity of ELISAs, providing a tool for the early and accurate diagnosis based on clinical samples and for epidemiological studies of SARS-CoV-2 [79, 80]. In addition, the NP-monoclonal antibody system and related test strips can be utilized for large-scale screening of COVID-19 samples [81].

### Multimeric neutralizing antibodies targeting multiple regions of SARS-CoV-2 proteins

SARS-CoV-2 nAbs can also be constructed by combining two or more neutralizing antibodies specific to SARS-CoV-2 proteins and/or antibody fusion with the receptor ACE2. The resultant multimeric (dimeric, trimeric, or tetravalent) nAbs may bind to their respective regions with neutralizing activity. A dimeric nAb, 89C8-ACE2, was designed by combining the SARS-CoV-2 NTD-targeting mAb 89C8 and the ACE2 ectodomain [61]. This nAb binds to the SARS-CoV-2 S protein RBD, inhibiting the S1–ACE2 interaction; it also neutralizes pseudotyped and authentic SARS-CoV-2 infection in vitro.

### Neutralizing antibodies to prevent and treat COVID-19 in humans

A variety of non–SARS-CoV-2-specific nAbs, such as anti-CD6 (itolizumab), anti-IL-6 receptor (tocilizumab), anti-IL17A (ixekizumab), and anti-C5 complement antibodies, have been used in patients with COVID-19, with the purpose of reducing or eliminating SARS-CoV-2–induced cytokine storms and inflammatory responses or treating SARS-CoV-2–related pneumonia [54, 82–93]. By comparison, only a few SARS-CoV-2-specific human nAbs, including REGN-COV2, LY-CoV555, LY-CoV016, AZD7442, AGD20, 47D11, 2B04, and CT-P59, have been tested in clinical trials and/or approved for emergency use to prevent and/or treat COVID-19 disease in humans [94–98]. Almost all of the nAbs under clinical trials target the RBD of SARS-CoV-2.

REGN-COV2, a cocktail of two SARS-CoV-2 RBD-targeting nAbs, REGN10933 (casirivimab) and REGN10987 (imdevimab), has been studied in a phase 1–3 clinical trial for outpatients with COVID-19, and the treatment resulted in reduced viral load without notable safety problems [94]. A phase 3 trial with subcutaneous administration of this nAb combination showed prevention of COVID-19 progression from asymptomatic to symptomatic disease in early SARS-CoV-2 infection [99]. In addition, patients with mild-to-moderate COVID-19 exhibited reduced hospital utilization after receiving REGN-COV2 treatment within a few days of symptom onset, and a low-dose REGN-COV2 infusion has been shown to improve COVID-19 symptoms [100, 101]. In addition to treatment, REGN-COV2 has been tested for the possibility of preventing SARS-CoV-2 infection. Subcutaneously injected REGN-COV2 prevented SARS-CoV-2 infection and the presence of COVID-19 symptoms in high-risk individuals who had close contact with SARS-CoV-2-infected persons [102].

LY-CoV555, an RBD-specific nAb also known as LY3819253 or bamlanivimab, has been evaluated in treating outpatients with COVID-19, and a phase 2 trial of bamlanivimab concluded that it decreased the viral load in these patients [95, 103]. In a phase 2/3 clinical trial, combinatorial treatment with bamlanivimab and another RBD-specific nAb, LY-CoV016 (also known as LY3832479, etesevimab, or CB6), reduced the viral load in nonhospitalized patients with mild-to-moderate COVID-19 illness, whereas there was no significant reduction in the viral load with monotherapy bamlanivimab treatment [97]. However, when coinjected with remdesivir, bamlanivimab does not exhibit therapeutic efficiency in patients hospitalized with COVID-19 [104]. As prophylactic prevention, bamlanivimab has been shown to reduce the incidence of COVID-19 among residents and staff of skilled nursing and assisted living facilities in a randomized phase 3 trial [105].

Clinical trials based on other SARS-CoV-2 RBD-targeting nAbs are ongoing. A phase 3 trial (NCT04723394) was conducted for coadministration with AZD7442 (a nAb combination of AZD8895 + AZD1061) to assess its safety and efficacy for the treatment of outpatient adults with COVID-19 and prevention of severe COVID-19 or death. Two additional phase 3 trials (NCT04625725 and NCT04625972) are planned to evaluate the safety and efficacy of preexposure and postexposure prophylaxis of this mAb combination in adults with COVID-19. In addition, a phase 2/3 trial (NCT04859517) has recruited to determine the efficacy and safety of the nAb AGD20 in the prevention (preexposure and postexposure prophylaxis) of COVID-19, and a phase 2/3 trial (NCT04805671) is planned to evaluate the efficacy and safety of this nAb in treating ambulatory participants with mild or moderate COVID-19. 47D11 and 2B04 nAbs have been used as monotherapy or combinatorial therapy in a phase 1 trial (NCT04644120) to evaluate their safety, pharmacokinetics, and pharmacodynamics in adults with COVID-19, whereas the nAb CT-59 has been applied in a phase 2/3 trial (NCT04602000) to evaluate its therapeutic efficacy and safety in outpatients with mild-to-moderate symptoms of SARS-CoV-2 infection.

Currently, bamlanivimab, etesevimab, and REGN-COV2 have been approved via Emergency Use Authorization (EUA) by the U.S. FDA for the therapeutic treatment of mild-to-moderate COVID-19 in adults and pediatric patients (≥12 years of age) from high-risk populations [106–109]. Moreover, the EUA also limits the use of these nAbs to treat only individuals who are hospitalized due to COVID-19 or other health concerns.

### Potential challenges and improvement of SARS-CoV-2-specific nAbs

Similar to other coronaviruses, including SARS-CoV-1 and MERS-CoV, SARS-CoV-2 has continually developed mutations during the COVID-19 pandemic, and different mutants have been found in all four structural proteins and other viral proteins [110]. Multiple strains that are variants of concern (VOCs) (B.1.1.7, B.1.351, P.1, and B.1.617.2) have been identified that carry single or combined amino acid mutations or deletions, including K417N/T, E484K, N501Y, L452R, and T478K in the RBD; L18F, T20N, P26S, 69-70del, D138Y, 144del, 156-157del, R158G, R190S, and 241-243del in the NTD; and D614G in S1 of the S protein [111, 112]. In addition, variant of interest (VOI) strains (B.1.427, B.1.429, B.1.525, B.1.526, B.1.617.1, B.1.617.3, and P.2) have also been defined, which carry mutations, such as L452R, S477N, E484K, and E484Q in the RBD; 69-70del, T95I, G142D, 144del, W152C, E154K, F157S, and D253G in the NTD; and D614G in S1 of the S protein [111, 112]. Among these variants, P.1 and P.2 belong to the B.1.1.28 lineage. These VOC and VOI strains may exhibit enhanced viral replication and/or transmission. Furthermore, other mutations that could cause escape from antibody recognition have been found in the S protein RBD or NTD regions through mapping, recombinant chimeric VSV/SARS-CoV-2 reporter viruses, polyclonal human plasmas antibodies, and other approaches [113–115]. Mutations in the SARS-CoV-2 RBD or NTD may lead to escape from antibody recognition and decreased neutralizing activity of nAbs against virus infection [113].

Many of the currently available nAbs specific to the prototypic SARS-CoV-2, including those targeting the NTD or RBD of the S protein, have reduced neutralizing activity against newly emerging SARS-CoV-2 variants with substitutions in the NTD or RBD, particularly when they are used alone [41, 116–120]. For example, the RBD-targeting nAbs 2-15 and C121 exhibit a complete loss of neutralizing activity against the P.1 variant [121]. Neutralization of the B.1.1.7 variant by the RBD-targeting nAbs COVA2-15, COVA1-18, and S309 has also been found to be reduced, which might be potentially due to the N501Y mutation [119]; nevertheless, this mutation has minimal effect on the neutralizing activity of the nAbs IgG ab1 and V_H_-Fc ab8 [122]. In contrast to RBD-specific nAb ab6, which can neutralize a pseudotyped SARS-CoV-2 variant containing a triple mutation (K417N, E484K, N501Y), the nAbs COV2-2196, COV2-3025, COV2-2381, and S2E12 exhibit reduced neutralizing ability against the B.1.351 variant containing the E484K and N501Y mutations [117, 123]. Notably, the RBD-targeting nAbs COV2-2050, 1B07, COVOX-384, and S2H58 exhibit loss of neutralizing activity against the Wash SA-B.1.351 variant containing the full S sequence of the South African strain [117]. In addition, the NTD-specific nAbs COV2-2676 and COVD-2489 also exhibit loss of neutralizing activity against the Wash SA-B.1.351 variant [117]. Other NTD-specific nAbs, such as 5-24 and 4-8, show completely abolished neutralizing activity against the B.1.351 variant, whereas the 159 nAb exhibits reduced neutralizing ability against the P.1 variant, potentially due to the mutations in the residues recognized by this nAb (L18F, T20N, P26S, D138Y, and R190S) [121, 124].

Notably, clinically approved RBD-targeting nAbs, including LY-CoV555, LY-CoV016, REGN10933, and AZD8895, exhibit abolished neutralizing activity against the SARS-CoV-2 P.1, B.1.351, or B.1.1.7 VOCs in vitro [121, 124]. This phenomenon could be explained by the fact that these nAbs are sensitive to mutations at residues 417, 484, and/or 501 of the SARS-CoV-2 RBD [124, 125]. In addition, LY-CoV555 and its combination with LY-CoV016 have been shown to exhibit reduced or loss of protective activity against VOCs, including B.1.351 and/or B.1.128, in hACE2-Tg mouse models [125]. A combination of clinically trialed nAbs, 2B04/47D11, have been shown to have decreased protective efficacy against the B.1.1.7, B.1.351, and B.1.128 variant strains in hACE2-Tg mice, and the protection was dose-dependent [125]. Apparently, the loss of neutralizing activity or protection by clinically approved nAbs against SARS-CoV-2 VOC strains will significantly affect the clinical use of these nAbs to treat COVID-19 patients, particularly when persons are infected with variants carrying related mutations. It is plausible to identify the neutralizing activity of nAbs approved via EUA against all potential VOC and VOI variants, test their combinatorial effects with other appropriate nAbs, or optimize injection dosages before use for clinical treatment of COVID-19 patients.

nAbs are engineered or fused to improve neutralizing activity to prevent and treat infection caused by SARS-CoV-2 mutant strains. Remarkably, combinatorial nAbs can be constructed by fusing the heavy chain (HC) of one nAb with the engineered light chain (LC) of another nAb targeting conserved epitopes on the NTD, RBD, or S2 regions of the S protein of multiple SARS-CoV-2 strains. It has been shown that the RBD-targeting nAb 222 with somatic mutations at the LC (222LC) maintains neutralizing activity against P.1, B.1.1.7, and B.1.351 variants and that a chimeric nAb (150HC/222LC) fused to the LC of the nAb 222 with the HC of another RBD-targeting nAb (150) restores or improves neutralization potency of the naive nAb against these SARS-CoV-2 VOC strains [124]. In addition, cocktail treatments combining nAbs recognizing epitopes on the NTD, RBD, or S2 or different epitopes on the same fragments are expected to exhibit synergistic effects and reduce the prevalence of antibody escape mutants, potentially improving nAb efficacy in preventing COVID-19. Indeed, NTD-targeting nAbs may neutralize mutant viruses that exhibit RBD-targeting antibody escape, and vice versa [54]. A combination of RBD- and NTD-targeting nAbs limits the development of escape mutants [126]. In addition, combinations of two RBD-targeting nAbs (such as REGN10933 and REGN10987), particularly three noncompeting RBD-specific nAbs (such as REGN10933, REGN10987, and REGN10985), improve the efficiency of individual nAbs in neutralizing SARS-CoV-2 VOC and VOI strains, preventing the development of escape mutants [127]. Moreover, treatment of hACE2-Tg mice, 129S2 immunocompetent mice, and hamsters with combinations of RBD-targeting nAbs, such as S309/S2E12, COV2-2130/COV2-2196, and/or REGN10933/REGN10987, prevents infection with multiple SARS-CoV-2 variants, including B.1.1.7, B.1.351, B.1.128, and SARS-CoV-2 containing N501Y/D614G mutations [125]. Crystal and cryo-EM structural analyses of the binding between nAbs and the SARS-CoV-2 S protein, its functional fragments, or other proteins provide an effective tool to rapidly identify novel and conserved neutralizing epitopes across SARS-CoV-2 variants, which will allow for the design of innovative countermeasures.

## Conclusions and prospects

The global COVID-19 pandemic has promoted rapid development and human testing of nAbs to prevent and treat SARS-CoV-2 infection. Among all of the SARS-CoV-2 proteins, S, particularly its RBD fragment, is the major target for the development of potent COVID-19 nAbs, and nAbs targeting this region are generally more potent than those targeting other regions, including the NTD. Most of these nAbs have been evaluated preclinically and exhibit neutralizing activity against SARS-CoV-2 in vitro and/or prophylactic or therapeutic protection of animals from SARS-CoV-2 challenge. Some of these nAbs also prevent infection or can be used to treat SARS-CoV-2-infected individuals. Nevertheless, the emergence of mutations in the antibody target regions, particularly in the S protein RBD, combined with the presence of antibody escape variants, makes it essential to design monovalent and multivalent nAbs or nAb cocktails with improved neutralizing activity and protective efficacy. Moreover, it would be advantageous to generate large amounts of monoclonal antibodies with relatively low cost for clinical use. Hopefully, new nAbs with broad-spectrum neutralizing activity against multiple SARS-CoV-2 variants and escape mutant strains will be developed, and more cost-effective manufacturing practices will be established. Ideally, these nAbs will provide safe and effective prophylactic and therapeutic agents that can be used to prevent and treat infection caused by current SARS-CoV-2 strains and future variants that may develop.
